# Developmental Profile of the Aberrant Dopamine D2 Receptor Response in Striatal Cholinergic Interneurons in DYT1 Dystonia

**DOI:** 10.1371/journal.pone.0024261

**Published:** 2011-09-02

**Authors:** Giuseppe Sciamanna, Annalisa Tassone, Giuseppina Martella, Georgia Mandolesi, Francesca Puglisi, Dario Cuomo, Grazia Madeo, Giulia Ponterio, David George Standaert, Paola Bonsi, Antonio Pisani

**Affiliations:** 1 Department of Neuroscience, University “Tor Vergata”, Rome, Italy; 2 Laboratory of Neurophysiology and Plasticity, Fondazione Santa Lucia I.R.C.C.S., Rome, Italy; 3 Department of Neurology, Center for Neurodegeneration and Experimental Therapeutics, University of Alabama at Birmingham, Birmingham, Alabama, United States of America; University of Chicago, United States of America

## Abstract

**Background:**

DYT1 dystonia, a severe form of genetically determined human dystonia, exhibits reduced penetrance among carriers and begins usually during adolescence. The reasons for such age dependence and variability remain unclear.

**Methods and Results:**

We characterized the alterations in D2 dopamine receptor (D2R) signalling in striatal cholinergic interneurons at different ages in mice overexpressing human mutant torsinA (hMT). An abnormal excitatory response to the D2R agonist quinpirole was recorded at postnatal day 14, consisting of a membrane depolarization coupled to an increase in spiking frequency, and persisted unchanged at 3 and 9 months in hMT mice, compared to mice expressing wild-type human torsinA and non-transgenic mice. This response was blocked by the D2R antagonist sulpiride and depended upon G-proteins, as it was prevented by intrapipette GDP-β-S. Patch-clamp recordings from dissociated interneurons revealed a significant increase in the Cav2.2-mediated current fraction at all ages examined. Consistently, chelation of intracellular calcium abolished the paradoxical response to quinpirole. Finally, no gross morphological changes were observed during development.

**Conclusions:**

These results suggest that an imbalanced striatal dopaminergic/cholinergic signaling occurs early in DYT1 dystonia and persists along development, representing a susceptibility factor for symptom generation.

## Introduction

DYT1 dystonia is a common form of inherited, generalized dystonia, with onset in childhood or adolescence, characterized by sustained muscle contractions causing twisting, repetitive movements and progressive abnormal postures [1]. It is produced by a single pathogenic codon deletion in the C-terminal of the protein torsinA, a member of AAA+ (“ATPases associated with a variety of cellular activities”) protein family of chaperone-like proteins, involved in protein trafficking, membrane fusion and participating in secretory processing [2]–[4]. Although at system level the precise consequences of torsinA mutation are still unclear, imbalances in neurotransmission in basal ganglia circuits as well as in the sensorimotor cortex and cerebellum have been documented [5]–[7].

Experimental and clinical evidence point to the striatum, where dopamine (DA) and acetylcholine (ACh) interact, as one of the principal sites of network dysfunction in DYT1 dystonia [8]–[11]. Although small in number, cholinergic interneurons are the sole source of striatal ACh, playing an essential role both in striatal synaptic plasticity and motor learning, as well as in the pathophysiology of movement disorders, such as Parkinson's disease (PD) and dystonia [12]–[15]. Of note, cholinergic interneurons are exclusively found in regions with a dense dopaminergic innervation, such as the dorsal striatum. In these regions, dopaminergic afferents exert a powerful control over cholinergic transmission. Maintenance of ACh levels is indeed regulated by ACh degrading enzymes, by muscarinic M_2_/M_4_ autoreceptors [14], [16] but also by an inhibitory DA D2 receptor (D2R) action [17]–[19]. Our previous studies identified a fundamental alteration in the balance between striatal DA and ACh. Normally, activation of D2Rs reduces the activity of cholinergic interneurons, whereas in interneurons from transgenic mice overexpressing human mutant torsinA (hMT), D2R activation dramatically increases, rather than decrease, the spike rate [20].

Because DYT1 dystonia is considered a neurodevelopmental disorder [2], [7], [9], in the present work we characterized the receptor and post-receptor mechanisms involved in this abnormal response, extending this analysis to different developmental stages. Similarly, the profile of High-Voltage-Activated (HVA) calcium currents was determined in the three strains of mice. Since strong level of torsinA mRNA is observed in cholinergic interneurons from both human and rodent striatum [21]–[23] a morphological analysis of cholinergic interneurons was performed at different ages. Our results demonstrate that torsinA mutation alters striatal function early in development. An imbalanced interplay between striatal DA and ACh distorts network function, and might predispose gene mutation carriers to develop dystonia, but these data also suggest that additional factors are required to trigger the appearance of clinical symptoms.

## Methods

### Transgenic animals and tissue preparation

Treatment and handling of mice were carried out in accordance with both the EC and Italian guidelines (86/609/EEC; D.Lvo 116/1992), approved by the Animal Ethics Committee of the University of Rome “Tor Vergata” (n. 153/2001A) and by the Santa Lucia Foundation Animal Care and Use committee (Authorization n 9/2006-A; protocol n. 14/2008 and n. 07/2011). All the efforts were made to minimize the number of animals utilized and their suffering. Transgenic mice were generated as previously described [24] and bred at our animal house. Non-transgenic (NT) littermates were utilized as controls. DNA extracted from mouse tail using the Extract-N-Amp Tissue PCR Kit (Catalog Numbers XNAT2, Sigma-Aldrich, Italy) was used for genotyping as described previously [6]. To amplify a 560 bp segment, two specific primers for human torsinA were utilised (5′-CACATTGCACTTTCCACATGCT -3′ and 5′-GTTTTGCAGCCTTTATCTGA-3′), and prepared according to the “Tissue Preparation” and “Reagent Preparation” protocols (Sigma-Aldrich; 35 cycles; annealing temperature 60°C). The human torsinA coding sequence was identified via 1.5% agarose gel electrophoresis. The mouse genotype was confirmed by restriction digestion with BseRI (New England BioLabs, USA). The human torsinA and human mutant torsinA PCR products were digested with BseRI into different fragments (279 bp, 238 bp, 24 bp, 22 bp and 279 bp, 259 bp, and 22 bp, respectively). Then, fragment profiles were identified by gel electrophoresis, using 2% SYBR Safe agarose (Invitrogen, Italy). Mice displayed comparable increases of torsinA protein in the striatum compared to their non-transgenic littermates [11].

#### Striatal slices

Mice were sacrificed by cervical dislocation under ether anaesthesia and the brain immediately removed from the skull. Coronal corticostriatal slices (250–300 µm) were cut with a vibratome in Krebs' solution (in mM: 126 NaCl, 2.5 KCl, 1.3 MgCl_2_, 1.2 NaH_2_PO_4_, 2.4 CaCl_2_, 10 glucose, 18 NaHCO_3_), bubbled with 95% O_2_/and 5% CO_2_. After 1 h recovery, a single slice was transferred into a recording chamber (∼0,5–1 ml volume), continuously superfused with oxygenated Krebs' medium, at 2.5–3 ml/min and maintained constantly at 32–33°C [6]. A single slice was used for pharmacological experiments.

### Electrophysiology

Cholinergic interneurons were visualized on a monitor (Sony) using a differential interference contrast (DIC, Nomarski) optical system combined with an infrared (IR) filter, and a monochrome CCD camera (C3077, Hamamatsu). Recordings were made with Multiclamp 700b and AxoPatch 200 amplifiers (Axon Instruments), using borosilicate glass pipettes (1.5 mm outer diameter, 0.86 inner diameter) pulled on a P-97 Puller (Sutter Instruments). Pipette resistances ranged from 2.5 to 5 MΩ. For voltage-clamp experiments, the internal solution consisted of (in mM): K^+^ -gluconate (125), NaCl (10), CaCl_2_ (1.0), MgCl_2_ (2.0), 1,2-bis (2-aminophenoxy) ethane-N,N,N,N-tetra-acetic acid (BAPTA) (0.1), Hepes (10), GTP (0.3) M_g_ -ATP (2.0); pH adjusted to 7.3 with KOH. All experiments were performed in presence of TTX (1 µM).

Current-clamp recordings were made in the perforated-patch configuration. Gramicidin was used as the pore-forming agent and was added to the pipette solution at an approximate concentration of 20 µg/ml. The tip of the pipette was filled with gramicidin-free intracellular solution. The perforation process was considered complete when the amplitude of the action potentials was steady and >60 mV, and electrode resistance was steady and <50 MΩ. For experiments with intrapipette BAPTA (10 mM) and GDP-β-S, current-clamp experiments were performed in the whole-cell configuration. Current–voltage relationships were obtained by applying 50 pA steps in both depolarizing and hyperpolarizing direction (from −500 to 500 pA, 600 ms).

### Statistical analysis

Data were analyzed using pClampfit 9.2 (Molecular Device), Origin 8.0 (Microcal) and Prism 5.0 (GraphPad). Numerical data are presented as means ± SEM. The evaluation of statistical difference was performed with two-way ANOVA test and paired parametric statistical test (Student's paired *t*-test). Nonparametric post hoc comparisons were also performed among groups using Tukey test to identify significant differences. Alpha was set at 0.01. The significance level was set at *p*<0.05.

### Acutely dissociated neurons

Striatal slices (∼450 µm) were incubated in Hepes-buffered Hank’s balanced salt solution (HBSS), bubbled with 100% O_2_, at 32°C. One slice was then incubated in HBSS media with 0.5 mg/ml protease XIV. After repeated wash-out in HBSS, the tissue was mechanically triturated with a graded series of fire-polished Pasteur pipettes. The cell suspension was placed in a Petri dish mounted on the stage of an inverted microscope (Nikon Diaphot, Japan). Healthy cells were allowed to settle for about 10-15 min [20], [25].

#### Electrophysiology

Patch-clamp recordings were performed in the whole-cell configuration from dissociated interneurons, by using glass pipettes (WPI PG52165-4, Germany) pulled with a Flaming-Brown puller (Sutter Instrument, Novato, CA, U.S.A.) and fire-polished before use. Pipette resistance ranged from 3 to 8 MΩ. The composition of the internal solution was (in mM): N-methyl-d-glucamine (185); HEPES (40); EGTA (11); MgCl_2_ (4); phosphocreatine (20); adenosine triphosphate, ATP (2 to 3); guanosine triphosphate, GTP (0 to 0.2); leupeptin (0.2); pH 7.36, 280 mOsm/L. After obtaining the cell access, cells were bathed in (mM): TEA-Cl (155); CsCl_2_ (5); HEPES (10); and BaCl_2_ (5) as the charge carrier; pH 7.35; 300 mOsm/L. Control and drug solutions were applied with a linear array of six gravity-fed capillaries positioned 500–600 µm close to the patched neuron. Recordings were made with an Axopatch 1D (Axon Instruments). Electrode resistances in bath were ∼3–6 MΩ. After formation of a GΩ seal and subsequent cell rupture, series resistance was compensated (75–85%) and constantly monitored. Data were low-pass filtered (corner frequency, 5 KHz). For data acquisition and analysis, pClamp 9.2 software (Axon Instruments) was used. Total HVA Ca^2+^ current was examined by utilizing ramp test (from −70 mV to +40 mV) or test pulse protocols (either a single step from −60 mV to +10 mV or incremental 10 mV steps from −70 mV to +40 mV). The different components of HVA Ca^2+^ currents (L-, N-, P-, and Q-type) were pharmacologically isolated by sequentially applying the selective channel blockers: nifedipine (NIFE, 5 µM), ω-Conotoxin GVIA (Ctx-GVIA, 1 µM), ω-agatoxin IVA (Atx-IVA, 20 nM), and ω-Conotoxin MVIIC (Ctx-MVIIC, 100 nM). The voltage dependence of activation was determined from measurements of tail current amplitude, which was expected to reflect the fraction of calcium channels opened during the preceding depolarization.

#### Data analysis

Voltage-activated currents were leak subtracted. Cells exhibiting leak currents >10 pA were not included in the analysis. The stability of cell capacitance (C_m_) during the experiment was monitored using the automated function of the Axopatch amplifier. Statistical analysis was performed using Microcal Origin (OriginLab, Northampton, MA, U.S.A.) or GraphPad Prism (GraphPad Software, San Diego, CA, U.S.A.) software. T-test or Mann-Whitney test were used for assessing statistical significance where appropriate. Multiple groups were compared using one-way or two-way ANOVA. The Tukey HSD test was used for post hoc comparison of the ANOVA. Values were considered statistically significant when p < 0.05 with α  = 0.001. Values given in the text and figures are mean ± SD of changes in the respective cell populations.

### Drug source

Nifedipine, ω-conotoxin GVIA, ω-conotoxin MVIIC, and ω-agatoxin IVA were from Tocris-Cookson, UK. All other compounds used were purchased from Sigma-Aldrich, Italy.

### Immunohistochemistry and imaging

Mice were deeply anesthetized (avertin, 0.002 ml/0.01 kg) and perfused through the aorta with ice-cold 4% paraformaldehyde. The dissected brain were equilibrated with 30% sucrose overnight. Thirty micrometer-thick coronal sections were cut with a freezing microtome and dehydrated through a graded ethanol series (50%–70%–50%). Slices were preincubated with 10% normal donkey serum solution (NDS) in PBS 0.25% Triton X-100 (TPBS) for 1 h at room temperature (RT) and incubated with the following primary antibodies at +4°C for three overnights: rabbit polyclonal anti-torsinA antibody 1∶600 (AbCam cod. ab34540) and goat anti-ChAT 1∶300 (Novus biologicals cod. NBP1-30052) in TPBS 1% NDS. After being washed with TPBS, the sections were incubated with Alexa488-conjugated donkey anti-rabbit (Invitrogen cod. A11008) and Cy3-conjugated donkey anti-goat secondary antibodies (Jackson Immuno Research cod. 705–165–147) 1∶200 for 2 h at RT. Sections were finally washed and mounted with Vectashield® mounting medium on poly-L-lysine-coated slides, air-dried, and coverslipped. The specificity of the antibody was previously tested [11]. All images were acquired with Zeiss LSM 700 confocal laser scanning microscope using the 40X/1.3 NA oil objective (resolution pixel 1024×1024). We collected several optical section images in the z-dimension (z-spacing, 1 µm) to fully capture the cholinergic interneurons. The confocal pinhole was kept at 1, the gain and offset setting were set to prevent saturation of the brightest signals, and sequential scanning for each channel was performed. To test for the specificity of the antisera, primary antibodies were substituted with normal serum and, accordingly, the immunolabeling was absent. Moreover, the specificity of torsinA antibody was already tested by western blot on striatum extracts (Napolitano et al., 2010). Images (five for each group and age) were acquired from serial coronal sections in the dorsal striatum of adult and P14 NT and hMT mice.

## Results

### Electrophysiological and morphological properties of cholinergic interneurons

**Figure 1:**
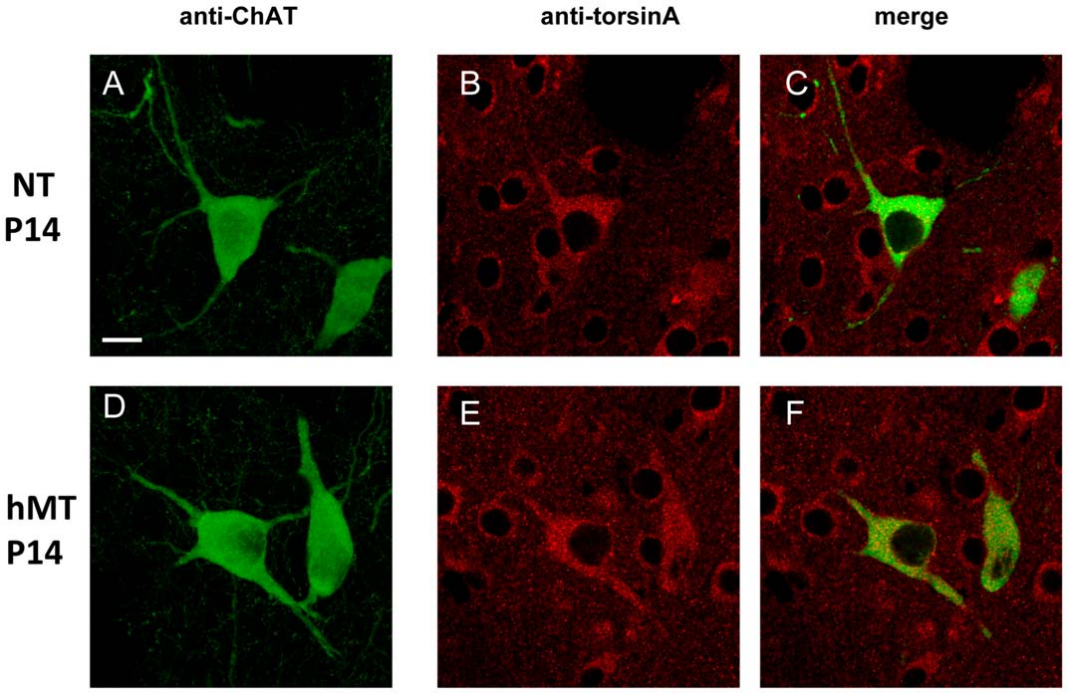
Immunohistochemical localization of torsinA in the cholinergic interneurons of NT and hMT mouse striatum. Representative confocal images (z-series projections) obtained from coronal slices of NT and hMT mice immunolabelled for ChAT (green, A, D). The macroscopic morphology of ChAT-positive cholinergic interneurons is apparently the same in the two groups at P14. Representative confocal images (single sections) showing the immunostaining of torsinA (red, B and E) in the ChAT-positive cholinergic neurons (merge, C and F) of NT (B–C) and hMT (E–F) mice. The cellular distribution of torsinA is similar between the groups. Scale bars: 10 µm.

TorsinA protein expression is temporally and spatially regulated in several regions of normal human and rodent brain [21], [23], [26]–[29]. In particular, torsinA expression, measured as mRNA level, is highest during prenatal and early postnatal development (until postnatal day 14, P14), suggesting a role in the developing nervous system [22], [27], [29]. Interestingly, among different neuronal populations, the striatal cholinergic interneurons seem to have a prominent level of torsinA at P14 [22]. In the transgenic hMT1 mice, the mutant torsinA transcript is under the regulation of a viral CMV promoter, and may have different spatial and temporal patterns of expression. Therefore, we investigated the immunohistochemical localization of torsinA in cholinergic interneurons of NT and hMT transgenic mice at this age. To this end, we performed double immunofluorescence experiments by incubating striatal coronal sections with specific antibodies against torsinA, and against choline acetyltransferase (ChAT) to detect cholinergic interneurons (Fig. 1). We observed a widespread and uniform torsinA labeling of the striatum at P14 in both NT and hMT (Fig. 1), as in the adult (data not shown; [6]). We did not observe macroscopic alterations in the cellular localization of torsinA in the hMT mice, which was confined to the cytoplasm and to proximal dendrites of the cholinergic neurons (Fig. 1B–C and E–F). Moreover, the cholinergic morphology defined by the ChAT-staining looked similar in both experimental groups at P14 (Fig. 1A–B) and at 3 months (data not shown). These results strengthen the observation that the pattern of torsinA distribution in NT and hMT mice is similar in striatal neurons, as it is in the striatum of normal and DYT1 mutation carriers [30].

**Figure 2:**
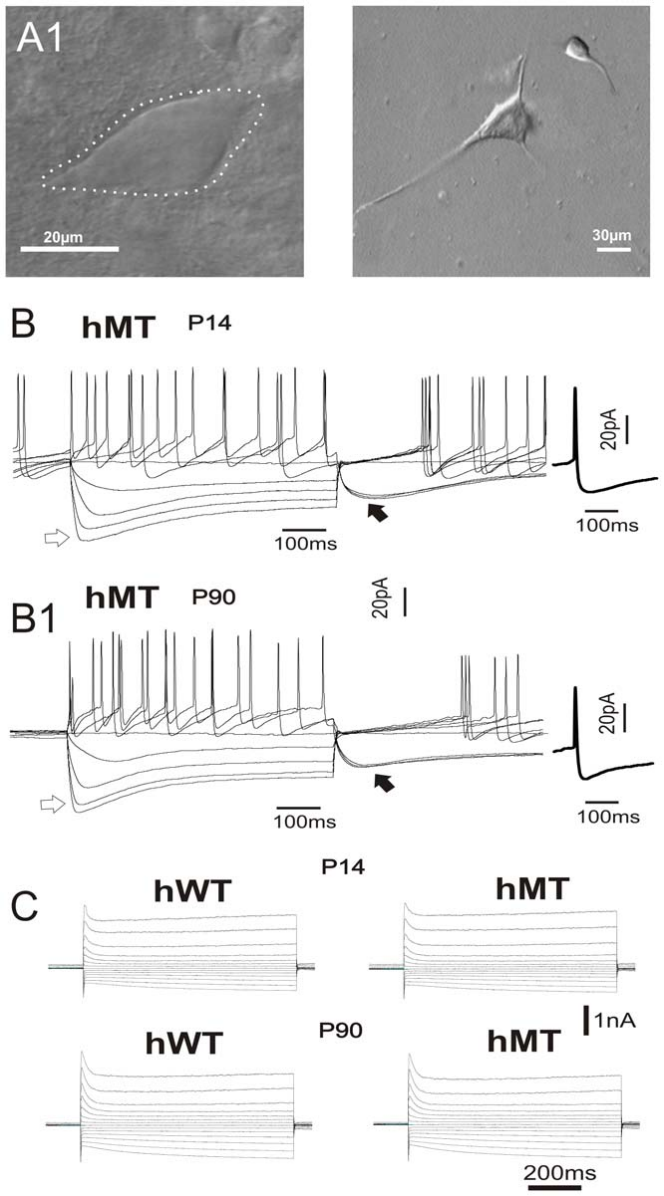
Basic electrophysiological properties of striatal cholinergic interneurons. Differential interference contrast images showing the morphological properties of striatal cholinergic interneurons in a slice preparation (A) and after enzymatic tissue dissociation (A1). Note the large, polygonal soma. B, B1. Representative traces showing voltage responses to current steps (100 pA, 600 ms) in both depolarizing and hyperpolarizing direction in cholinergic interneurons of hMT recorded at P14 and P90. Note the prominent *I_h_* current evoked by hyperpolarizing step (*white arrow*) and the robust AHP current following the step ending (*black arrow*). A single representative action potential is shown at higher sweep speed. C. I-V relationship (10 mV step, 600 ms) recorded in the voltage-clamp mode confirm that no significant difference in membrane properties were detected between hMT and hWT mice.

Cholinergic interneurons, visualized with IR-DIC videomicroscopy, exhibited typical morphological features such as a large polygonal cell body and two-three major aspiny dendritic branches (Fig. 2A–A1). The electrophysiological properties of these neurons confirmed the morphological identification. The recorded cells had a depolarized resting membrane potential (RMP), a long-lasting afterhyperpolarization (AHP) and a prominent I_h_ current elicited by hyperpolarizing current steps (Fig. 2B–C). All these properties have been unequivocally attributed to cholinergic interneurons [31], [32]. No significant difference of intrinsic membrane properties, such as RMP (−56±4.5 mV and −61±2.6 mV, at P14 and P90, respectively), input resistance (110±8.5 MΩ and 128±5.8 MΩ, at P14 and P90), I_h_ current (368±11 pA and 318±21 pA at P14 and P90) was recorded in hMT mice between P14 and P90 (Fig. 2; n = 23; p>0.05). Similarly, such properties did not differ among the three strains of mice (Table 1; p>0.05). In order to study the spontaneous firing activity of cholinergic interneurons, current-clamp experiments were performed in the perforated patch-clamp configuration, preserving a more physiological intracellular ion concentration with respect to the whole-cell configuration [33]. In such condition, tonic rhythmic spiking activity was observed in nearly all of the recorded neurons (∼90%).

**Table 1:** Intrinsic membrane properties of cholinergic interneurons in NT, hWT and hMT mice.

		NT			hWT			hMT	
	RMP (mV)	IR (MÙ)	I_h_(pA)	RMP (mV)	IR(MÙ)	I_h_(pA)	RMP (mV)	IR(MÙ)	I_h_(pA)
**P14**	−58±4	132±8	374±12	−56±3	134±6	361±15	−56±6	110±8	368±11
**P90**	−60±2	142±9	338±24	−59±5	161±9	380±8	−61±3	128±8	318±21

**Figure 3:**
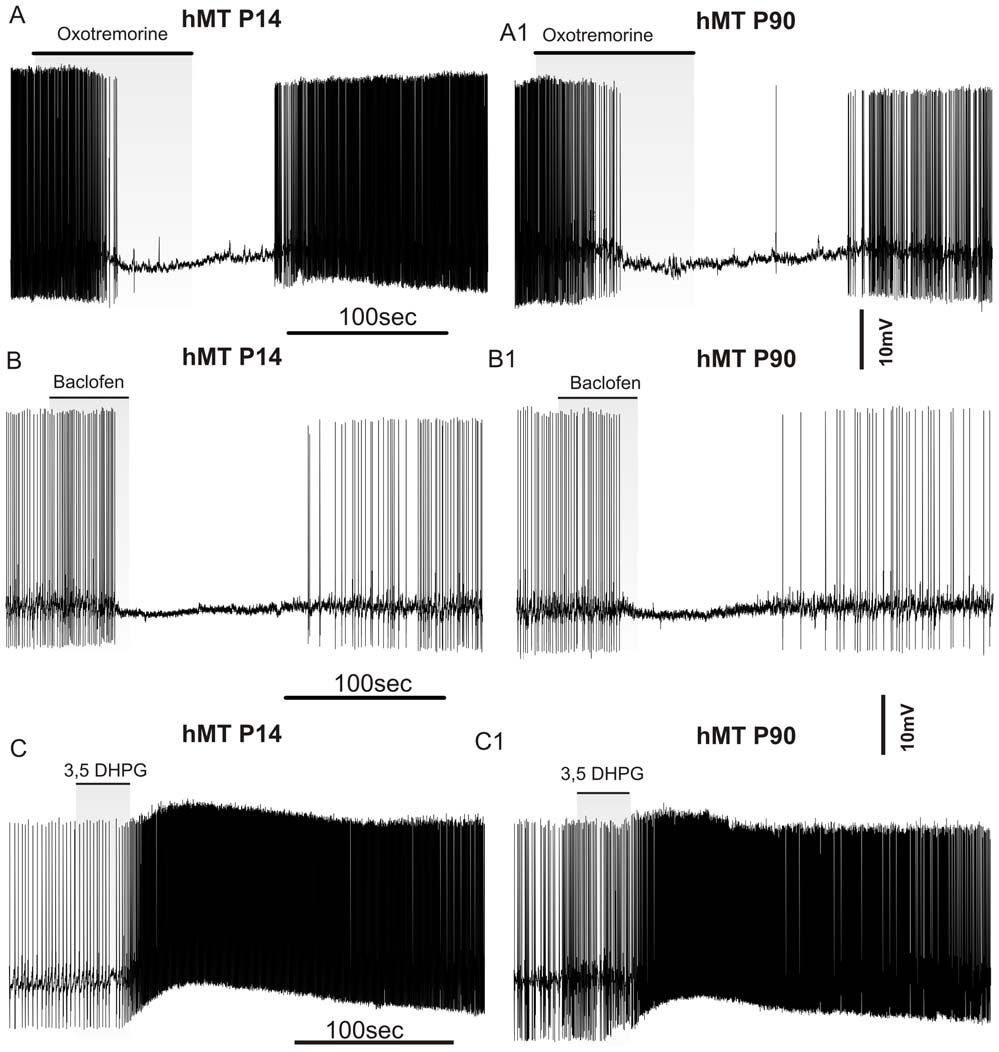
Cholinergic interneurons exhibit normal response to M_2_/M_4_, GABA_B_ and group I mGlu receptor activation. Representative traces of cholinergic interneurons recorded in the perforated patch-clamp configuration. Recordings were collected from hMT mice at P14 *(left)* and P90 *(right)*. A–A1. M_2_/M_4_ receptor activation by oxotremorine (300 nM, 3 min) induced a transient firing cessation with a membrane hyperpolarization. No significant differences were measured at both ages. B–B1. Similarly, application of Baclofen, a GABA_B_ receptor agonist (10 µM, 2 min) reversibly blocked firing activity in hMT mice. C–C1. Activation of group I mGlu receptors by 3,5-DHPG (10 µM, 30 sec) induced a prominent membrane depolarization coupled with a transient increase of firing rate.

Spontaneous firing activity of cholinergic interneurons is modulated by intrinsic membrane properties, but also by activation of distinct neurotransmitter receptors [34]. To control for non-specific effects of the torsinA overexpression, we investigated the effect of muscarinic, GABAergic and metabotropic glutamate receptor activation on the tonic activity of cholinergic interneurons. First, we measured the response to muscarinic M_2_/M_4_ autoreceptor activation at P14 and P90, which produces a membrane hyperpolarization coupled to a transient interruption of firing activity [16], [35]. Similarly to NT and hWT animals, bath-application of the selective M_2_/M_4_ agonist oxotremorine (300 nM, 3 min) caused a membrane hyperpolarization and inhibited neuronal firing in hMT mice at both ages examined (Fig. 3A,A1; P14: 2.3±0.81 mV; P90: 2.8±0.66 mV; p>0.05; n = 8 for each group). These data show that the muscarinic autoreceptor function is preserved in mice with mutant torsinA at both ages tested. No difference was measured among genotypes (not shown; n =  8; p>0.05). Next, we analyzed the response of cholinergic interneurons from hMT mice to baclofen, a GABA_B_ receptor agonist, which has been shown to inhibit cell firing [35]. Bath-applied baclofen (10 µM, 2 min) caused a reversible membrane hyperpolarization coupled to blockade of firing activity both in P14 (3.5±0.8 mV), as well as in P90 mice (2.8±0.5 mV, n = 8 for each group, p>0.05) (Fig. 3B,B1). The response to baclofen was not significantly different among NT, hWT and hMT neurons (not shown; n = 8 for each group; p>0.05). Finally, we examined the effect of dihydroxyphenylglycine (3,5-DHPG), a group I metabotropic glutamate receptor agonist, which has a robust excitatory effect on this cell type [36]. Bath-applied 3,5-DHPG (10 µM, 30 s) induced a rapid and transient membrane depolarization with a prominent increase in firing rate. The response to 3,5-DHPG was not significantly different between neurons recorded at P14 and P90 (Fig. 3C,C1; respectively from 0.5±0.27 to 7.38±2.3 Hz; and from 0.61±0.38 to 6.68±1.88 Hz; n = 8 for each group, p>0.05). No difference was measured among genotypes (not shown; n =  8; p>0.05).

### Early alteration of D2R signalling in cholinergic interneurons

In a previous report, we showed that application of the D2R agonist quinpirole induces a paradoxical excitatory effect in striatal cholinergic interneurons from 9-months-old hMT mice overexpressing human mutant torsinA [20]. In order to establish whether developmental changes affect D2R signalling, we extended this characterization to P14 and P90 hMT mice. The latter group is comprehensive of recordings performed between P70 and P120. Because no difference was observed, data were pooled together.

**Figure 4:**
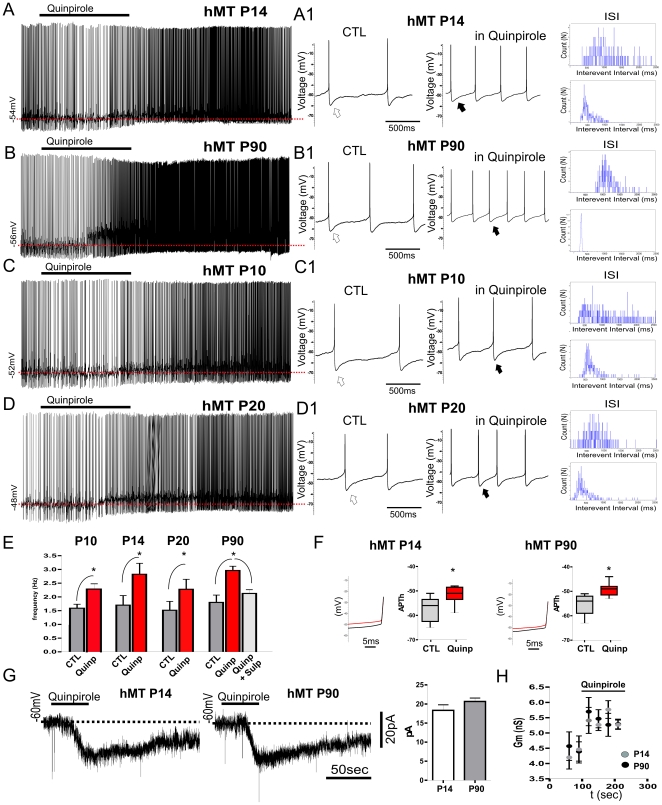
Early abnormal responsiveness to D2R stimulation in hMT mice. A–D. In hMT mice, quinpirole (10 µM, 2 min) caused a paradoxical membrane depolarization coupled to a large increase in the rate of action potentials in cholinergic interneurons recorded from mice at four different developmental ages. A1–D1. Sample traces show, at higher sweep speed, the firing rate measured before and at the peak of the effect of quinpirole application. Note the reduction of mAHP in quinpirole (white and black arrows). Left-shift of the ISI plot *(inset)* confirm the increased firing frequency induced by quinpirole at all tested ages. E. Graph plots summarize the effect of quinpirole at P10, P14, P20 and P90. The D2R antagonist sulpiride (3 µM) prevented the aberrant response to quinpirole. F. Zoom of action potential recorded in control conditions (black trace) and in the presence of quinpirole (red trace) at P14 and P90. Note the decrease of AP threshold induced by D2R activation. Graphs summarize the changes at both ages. G. Voltage-clamp recordings (holding potential −60 mV) showing that bath-application of quinpirole induces an inward current in hMT mice at P14 and P90. H. Time-course of quinpirole effect on the membrane conductance at P14 and P90. D2R activation induced a significant increase in membrane conductance at both ages.

Bath-application of quinpirole (10 µM, 2 min) had no significant effect on membrane potential or input resistance of interneurons recorded from NT and hWT mice at both ages examined, though a slight, but not significant decrease in firing discharge was observed (n = 8; p>0.05; Fig. S1A). Conversely, in hMT mice recorded at both P14 and P90, quinpirole depolarized the recorded cells and significantly increased the rate of action potential discharge (Fig. 4A.B; P14: from 1.6±0.35 to 2.7±0.25 Hz; P90: from 1.7±0.20 to 2.9±0.15 Hz, n = 10 for each group, p<0.05). Interspike interval (ISI) exhibits a clear left-shift of the histogram, confirming the rise in firing rate. In fact, after application of quinpirole, the number of action potentials within a short time interval increased (Fig. 4A1,B1). Pre-treatment with the D2R antagonist sulpiride (3 µM) fully prevented the response to quinpirole at both ages (Fig. 4E), confirming the selective activation of D2Rs. In principle, quinpirole can activate both D2Rs and D3Rs proteins. However, the dorsal striatum lacks D3Rs, and therefore the effects of quinpirole in this region are ascribed to D2Rs [37], [38].

**Figure 5:**
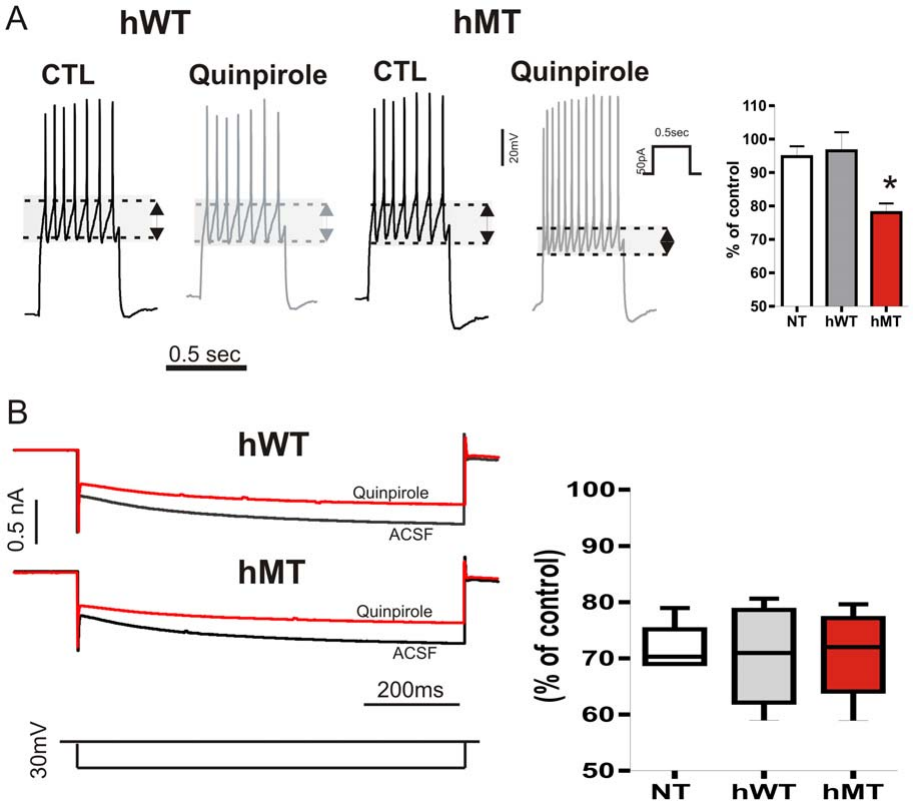
I_h_ and mAHP current modulation by D2R activation. A. Representative traces of mAHP recorded after a brief depolarizing current pulse (50 pA, 500 ms) in hWT and hMT mice. Note that in hMT mice, quinpirole application (gray trace) significantly reduced mAHP. Summary graph of D2R-mediated effects on mAHP in the three genotypes. B. Hyperpolarizing voltage steps (30 mV, 800 ms) in the presence of TTX (1 µM) were delivered to induce an I_h_ current. Representative traces recorded in whole-cell configuration before (black) and after (red) quinpirole application in hWT and hMT mice. Graph plot summarizes the effect of D2R activation on the I_h_ current in the three groups of mice.

Because developmental changes of both striatal cholinergic innervation as well as of torsinA expression occur during an early postnatal period [29], [39], we extended our electrophysiological analysis to two further ages: P10 and P20, in order to provide a more complete developmental profile. Consistently with data collected at P14 and P90, in interneurons recorded from hMT at P10 and P20, bath application of quinpirole (10 µM, 2 min) significantly depolarized the cells, increasing the rate of action potential discharge (Fig. 4C,D,E; P10: from 1.59±0.15 to 2.3±0.17 Hz; P20: from 1.53±0.33 to 2.30±0.35 Hz, n = 4 for each group, p<0.05). Moreover, in hMT mice D2R activation led to a significant change in the threshold for action potential discharge (AP_th_) at both ages, consistent with an increase in cell excitability (Fig. 4D: P14: from −57.3±1.4 to −51.50±1.06; P90: from −55.30±1.22 to −49.40±0.8; P<0.005; n = 10 for both groups; p<0.05). Finally, in voltage-clamp experiments, bath application of quinpirole (10 µM, 2 min) induced a long-lasting inward current (*I*_quinp_) in hMT mice at both ages tested (Fig. 4E: P14: 18.33±1.4 pA; P90: 20.67±0.88; n = 8 for each age; p<0.05). No significant change was observed between the two different ages (p>0.05). Examination of the changes in membrane conductance using hyperpolarizing voltage step (10 mV, 600 ms) elicited before and during quinpirole application, revealed that *I*_quinp_ was associated with a significant increase in membrane conductance in hMT mice at P14 and P90 (Fig. 4H; P14: at rest 4.20±0.38 nS, in quinpirole 5.27±0.15 nS; P90: at rest 4.57±0.47 nS, in quinpirole 5.30±0.16 nS; n = 6 p<0.05). During rhythmic spontaneous firing activity of cholinergic interneurons, individual action potentials are followed by a brief calcium-mediated current responsible for membrane hyperpolarization, termed medium afterhyperpolarization (mAHP) [40]. In hMT mice at both ages, D2R activation induced an evident reduction of this AHP (Fig. 4A1–B2). To better explore this effect, we performed current-clamp experiments using brief, small current pulses (50 pA, 500 ms), able to induce a series of action potentials (Fig. 5A). Then, we measured the mAHP evoked after each individual spike before and after application of quinpirole. In hMT mice, D2R activation clearly reduced the mAHP (Fig. 5A *right*; 78±2.7% of control; n = 8; p<0.05), whereas it failed to affect mAHP currents in hWT or NT mice (Fig. 5A *left*; NT: 94±3,5%; hWT: 96.8±5.5% of control, p>0.05). These results are consistent with the changes in action potential threshold, and indicate that during the abnormal D2R-activation, the smaller hyperpolarization enables a more rapid membrane repolarization, allowing the cells to fire at higher rate.

Finally, we tested the effect of D2R activation on I_h_ current, which participates in the modulation of firing activity [41]. In hMT mice, bath-applied quinpirole induced an inhibitory effect on the I_h_ current similar to that observed in interneurons from NT and hWT mice (Fig. 5B expressed as % of control, NT: 72.1±23.5; hWT: 70.4±5.0; hMT: 70.60±4.5; n = 6 for each group; p>0.05), thereby ruling out a major involvement of I_h_ current in the abnormal D2R-mediated response.

### Gi/Go proteins involvement in D2R-mediated response of hMT mice

**Figure 6:**
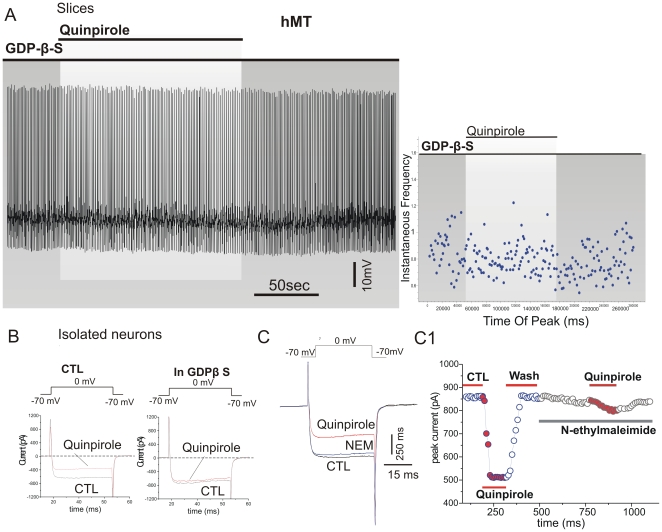
Gi/Go protein involvement in D2R-mediated response. A. Representative traces of a hMT interneuron recorded in whole-cell current-clamp configuration. In the presence of GDP-β-S in the recording pipette, the response to quinpirole was fully prevented. The graph shows that the instantaneous firing frequency did not change after bath application of quinpirole (10 µM, 2–4 min). B. Data were collected from acutely isolated interneurons of hMT mice. Pipette loading with GDP-β-S was able to block the inhibitory effect of quinpirole onto HVA calcium currents. C. Application of quinpirole (10 µM 2–4 min) produced a robust inhibition of the total HVA calcium currents evoked by voltage-ramp protocols. Time-course of drug application show that the pre-application of N-ethylmaleimide (NEM) fully occluded the inhibitory effect mediated by D2Rs activation. Blue circles represent the HVA currents recorded in control condition. Red circles report the inhibitory effect of quinpirole.

D2R signalling is mediated through activation of Gi/Go proteins, which are coupled to inhibition of adenylyl cyclase and ion channel modulation [19], [42]. To investigate whether the D2R-dependent signalling mechanisms were altered in hMT mice, recording pipettes were loaded with GDP-β-S (500 nM). The non-hydrolysable GDP-β-S competes with endogenous GTP for the nucleotide binding on G-proteins, locking G-proteins in an inactive state. In whole-cell current-clamp experiments, intracellular replacement with GDP-β-S fully prevented the response to quinpirole, which failed to alter RMP or to induce significant increase of firing rate in hMT (Fig. 6A) as well as hWT mice (Fig. S1B). Accordingly, recordings from dissociated striatal cholinergic interneurons showed that GDP-β-S was able to prevent, in hMT mice as well as in NT and hWT animals, the inhibitory action of quinpirole (10 µM) on HVA calcium currents (Fig. 6B; n = 13; p>0.05). In addition, the inhibitory effect of quinpirole on calcium currents was blocked by pre-application of N-ethylmaleimide (NEM, 50 µM Fig. 6C), a sulfhydryl alkylating agent, which inhibits G-proteins [19]. Taken together, these results suggest that the aberrant D2R response involves the coupling to their cognate G-proteins.

### Calcium ion involvement in D2R-mediated response of hMT mice

**Figure 7:**
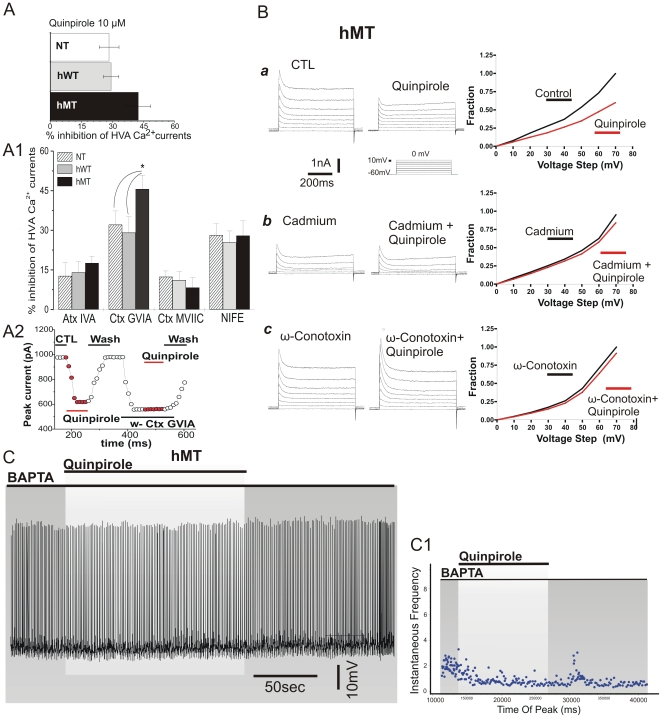
Calcium ion involvement in D2R-mediated response. A–A2. Data collected from acutely isolated neurons. A. Graph plot summarizes the inhibitory effect of quinpirole application on HVA calcium current evoked by voltage-ramp protocols. A1. Bar graph shows an increased Cav2.2/N-type fraction of total HVA current in isolated interneurons of hMT mice. A2. Time-course of the peak calcium current before and after drug application. The inhibitory effect produced by quinpirole was occluded by pre-application of ω-conotoxin GVIA. B. Data collected from brain slices. Representative traces of voltage dependent currents evoked by a series of depolarizing steps from an holding potential of −60 mV. Currents were evoked in the presence of TTX (1 µM) in order to eliminate Na_v_ channel-mediated inward currents in the sub- and supra-threshold voltage range. Application of the D2R agonist quinpirole (10 µM, a) clearly reduces the outward current evoked by depolarizing steps. Slices pre-incubation with CdCl_2_ (300 nM) almost fully abolished the quinpirole-mediated effect (b). Blockade of Cav2.2/N-type calcium channels by ω-conotoxin GVIA (1 µM, c) largely reduced the quinpirole effect on step-evoked current. Graphs report the normalized current recorded for each voltage level. C. Representative traces of cholinergic interneurons recorded in whole-cell current clamp experiments in hMT mice. Intracellular replacement with an high BAPTA concentration (10 mM) fully prevented the abnormal response after quinpirole application. C1. Plot of instantaneous firing frequency confirm that no significant increase of firing rate was caused by D2R activation in the presence of BAPTA.

D2R activation has been shown to inhibit calcium currents in this neuronal subtype [19]. In a set of experiments, we recorded calcium currents from acutely dissociated cholinergic interneurons to test the inhibitory action of quinpirole. In hMT mice at P90, the quinpirole-mediated inhibition on HVA current was significantly increased, as compared to NT and hWT mice (Fig. 7A; NT: 29.39±3.72%; hWT: 28.4±4.8%, hMT: 42.54±5.9%; n = 12 for each group; p<0.05). To investigate in detail the composition of the total HVA current in mutant mice, we performed a further set of experiments by sequentially applying selective calcium channel blockers in order to isolate each fraction. Application of nifedipine (NIFE, 5 µM, L-type calcium channel blocker), Ctx-MVIIC (100 nM, Q-type blocker) and Atx-IVA (20 nM, P-type blocker) produced a comparable effect in all the examined groups (Fig. 7A1; n = 10; p>0.05). Conversely, in hMT mice Ctx-GVIA (1 µM, Cav2.2/N-type blocker) inhibited a greater portion of total calcium current than in NT and hWT mice (Fig. 7A1; NT: 32.13±5.9%; hWT: 29.12±6.01%, hMT: 45.13±5.2%; n = 12; p<0.05). To investigate whether the altered contribution of Cav2.2/N-type fraction to total HVA current was involved in the aberrant D2R-mediated response of cholinergic interneurons, we performed occlusion experiments with each calcium channel blockers. Only Ctx-GVIA prevented the inhibitory effect of quinpirole (Fig. 7A2; quinpirole in Ctx-GVIA 102.3±12.4% of control; n = 8; p>0.05), demonstrating that the increased inhibitory effect of quinpirole on total HVA calcium current observed in hMT mice is entirely mediated by an increased contribution of Cav2.2/N-type channel fraction.

To further confirm these observations, a parallel set of voltage-clamp experiments was performed in slice preparations. Voltage-dependent currents were evoked by a series of depolarizing steps from an holding potential of −60 mV. Bath-application of quinpirole (10 µM) induced a transient current at depolarizing holding potentials in hMT mice (Fig. 7Ba). D2R activation, in fact, reduced the transient current evoked by a depolarizing step from −60 to +10 mV (Fig. 7Ba). To test whether the quinpirole effect was mediated by HVA calcium channels, the non-specific blocker cadmium (CdCl_2_, 300 nM) was applied to the perfusing solution. CdCl_2_ fully occluded the effect of quinpirole (Fig. 7Bb). Furthermore, as observed in the recordings from isolated cells, pre-incubation with Ctx-GVIA (1 µM) also prevented the effect of quinpirole on step-evoked whole-cell current (Fig. 7Bc), further supporting the hypothesis that the D2R action is mediated by Cav2.2/N-type channels.

Finally, the role of calcium ion was confirmed by loading the recording pipette recording with high concentration of the calcium chelator BAPTA (10 mM). In such experimental condition, in slices from hMT mice, quinpirole failed to induce the abnormal D2R-evoked current (Fig. 7C, n = 6, p>0.05).

### D2R-Adenosine A2A receptor interaction

Biochemical and electrophysiological data demonstrate that adenosine A2A receptors (A2ARs) are co-localized on the same striatal medium spiny projection neurons expressing D2Rs, where they counteract the activity of this latter class of receptors [11], [43]. More recently, a co-localization of A2ARs and D2Rs was demonstrated also in cholinergic interneurons [44]. This evidence prompted us to test if the defective D2R function could be overcome by blocking A2ARs. Pretreatment of striatal slices with the selective A2AR antagonist SCH58621 (50 nM, 15 min) did not affect basal intrinsic properties of cholinergic interneurons. In the presence of SCH58621 in the bathing solution, the excitatory effect of quinpirole (10 µM, 2 min) persisted unchanged, suggesting that the functional coupling of A2ARs and D2Rs is lost in this neuronal subtype in hMT mice (data not shown, n = 6, p>0.05).

## Discussion

Onset of DYT1 dystonia occurs typically between childhood and early adolescence, an observation consistent with the notion that it may be viewed as a neurodevelopmental disorder [2], [7], [9]–[10]. A peculiar feature of this inherited form of dystonia is represented by the reduced penetrance (∼30%), but it remains unclear what triggers dystonic symptoms in gene mutation carriers. If carriers do not show symptoms prior to 26 years of age, they usually remain unaffected. However, it is now well-established that non-manifesting gene carriers have endophenotypes even without overt dystonia, expanding the notion of penetrance and phenotype [45]. To this respect, the lack of clear-cut neurodegenerative alterations in post-mortem brain samples from DYT1 patients [46] highlights the relevance of an underlying network distortion [2], [6], [7]. In the present work, we demonstrate that the physiological balance between DA and ACh is disrupted as early as at P10 in mice with the DYT1 mutation and persists along different developmental stages, without gross morphological changes. This study represents the first systematic electrophysiological and morphological characterization of the DA/ACh interplay along development performed in an animal model of DYT1 dystonia.

Cholinergic interneurons are autonomous pacemakers, and their spiking activity, and consequently ACh release, are under the inhibitory control of both muscarinic M_2_/M_4_ autoreceptors and D2Rs [14], [16]–[19]. Here, we show that in mice with the DYT1 mutation, the M_2_/M_4_ muscarinic response is preserved, whereas conversely, the D2R-mediated inhibition is shifted towards excitation throughout different developmental stages.

Dysfunction of the striatal dopaminergic system in dystonia has been documented in a number of clinical and experimental settings [8], [9], [47]–[51]. Indeed, numerous imaging studies have identified D2R alterations in patients with primary dystonia [47], [52], [53]. Recently, Carbon and co-workers demonstrated a significant reduction of D2R availability in the caudate and putamen of both manifesting and non-manifesting DYT1 mutation carriers [9], postulating that these alterations might represent an endophenotype or a susceptibility factor for developing dystonia. Alterations in DA neurotransmission is evident also in models of DYT1 dystonia. Mice overexpressing mutant torsinA display an increased DA turnover [49], and an altered DA transporter function [54]. In addition, in striatal spiny projection neurons, a D2R dysfunction has been demonstrated, with reduced abundance of D2R protein and impaired capacity of D2Rs to activate their cognate Go/i proteins [11]. Nonetheless, it remains still to be addressed whether these alterations represent compensatory changes or if they may be considered pathogenic features of the disease.

Cholinergic interneurons express D2Rs [55] and our results with sulpiride confirm a receptor-specific alteration in D2R-mediated responses. These observations point directly to a decreased D2R function, although the molecular link between mutated torsinA and D2R dysfunction has not been identified yet [10]. Most of the effects exerted by D2Rs are mediated via activation of Gi/Go proteins, which are coupled to inhibition of adenylyl cyclase and modulation of ion channels [42], [56]. Our data show that the D2Rs retain their ability to activate G proteins in cholinergic interneurons. Indeed, intracellular application of the G protein inhibitor GDP-β-S, as well as bath-perfusion of the alkylating agent NEM, completely prevented the abnormal response to quinpirole. However, mutant mice display lower levels of striatal RGS9 [11]. This protein acts as GTPase-accelerating protein, thereby promoting G protein inactivation. One possible interpretation is that reduced RGS9 expression represents an adaptive response to compensate for reduced D2R functioning. Nonetheless, it is also plausible that decreased activity of RGS9 would enable a persistent coupling to D2R, prolonging its activation in a non-physiological manner.

This assumption finds support in our data on HVA calcium currents. D2R activation is coupled to inhibition of Cav2.2/N-type channels in a membrane-delimited, PKC-insensitive manner [19]. In cholinergic interneurons from hMT mice, the sensitivity to quinpirole-mediated inhibition of the total calcium evoked currents (HVA) was strikingly increased. Accordingly, these changes were accompanied by an enhanced representation of Cav2.2/N-type current fraction. Moreover, the D2R mediated response was fully prevented by blocking Cav2.2/N-type channels. Together with previous evidence demonstrating such alterations in 9-months-old mice [20], our current data testify the occurrence of these changes at early developmental stages. These data are even more intriguing considering that the D2R-dependent modulation of Cav2.2/N-type channels normally exhibits an age-dependent decrease in cholinergic interneurons. In fact, D2R-mediated inhibition decreases in parallel with the reduction in Cav2.2/N-type channel contribution to total current fraction [57], [58].

The functional increase of the Cav2.2 -mediated current fraction would be expected to enhance the inhibitory effect of quinpirole on cholinergic interneurons. Then, what drives the inhibition into a paradoxical excitation? A plausible explanation resides in the complex regulation of the pacemaking activity of these interneurons. Calcium currents are selectively coupled to calcium-dependent potassium conductances underlying the afterhyperpolarization (AHP) which follows single action potential [40]. In hMT mice, the membrane depolarization coupled to the increase in firing discharge induced by quinpirole was accompanied by a reduction in the mAHP amplitude, thereby moving cell membrane potential closer to firing threshold. Consistently, chelation of intracellular calcium by injecting BAPTA through the recording pipette completely abolished the effect of quinpirole, further suggesting that activation of calcium-dependent potassium conductances plays a role in the altered D2R-mediated response.

Our findings do not exclude the possibility that other neurotransmitter systems may be affected by mutant torsinA in other models and in humans. However, the normal responses to metabotropic glutamate receptor activation, together with the normal muscarinic autoreceptor and GABAB responses described in the present study seem to rule out a major role of other neurotransmitters.

Abnormal D2R signaling has been shown to have relevant functional consequences on striatal circuitry, by elevating the levels of ambient ACh. The increased cholinergic tone, proven by an enhancement of AChE activity and consistent with an enhanced turnover of ACh, was indeed responsible for the loss of corticostriatal long term depression and synaptic depotentiation in hMT mice [6]. These data are coherent with the clinical observation that an effective therapy for dystonia is treatment with anticholinergic drugs such as trihexyphenidyl, a preferred M_1_ muscarinic receptor antagonist [59], [60], and strengthens the hypothesis that the striatal DA/ACh imbalance has a relevant role in the pathophysiology of dystonia. Although the molecular mechanisms underlying torsinA dysfunction have not been fully elucidated, mutant torsinA might induce a sensitization to endoplasmic reticulum stressors. An abnormal protein trafficking would have functional consequences on neurotransmission, as changes in the levels of neurotransmitter transporters, receptors and accessory proteins, with profound effects on synaptic activity [10].

To date, the absence of a clear link between electrophysiological alterations and motor phenotype described in mice overexpressing mutant torsinA DYT1 mouse model suggest caution in the interpretation of our results. However, these data seem to suggest that approaches aimed at normalizing striatal dopaminergic/cholinergic balance may be a successful approach to developing alternative treatments for DYT1 dystonia.

## Supporting Information

Figure S1 Representative traces of cholinergic interneurons recorded in perforated patch-clamp mode. A. In hWT mice bath-application of quinpirole (10 μM 2-4 min) caused a small reduction of firing rate activity. Time-course plot *(inset)* shows that quinpirole induced a small reduction of instantaneous firing frequency. B. In hWT animals, D2R activation did not produce any significant effect on membrane potential or firing activity when GDP-β-S (10 mM) was added to the intracellular recording solution.
(TIF)
