# Supplementary figures and images for: Developmental Profile of the Aberrant Dopamine D2 Receptor Response in Striatal Cholinergic Interneurons in DYT1 Dystonia
Source: PLoS One. 2011 Sep 2;6(9):e24261. doi: 10.1371/journal.pone.0024261 (PMC3166312; doi:10.1371/journal.pone.0024261)

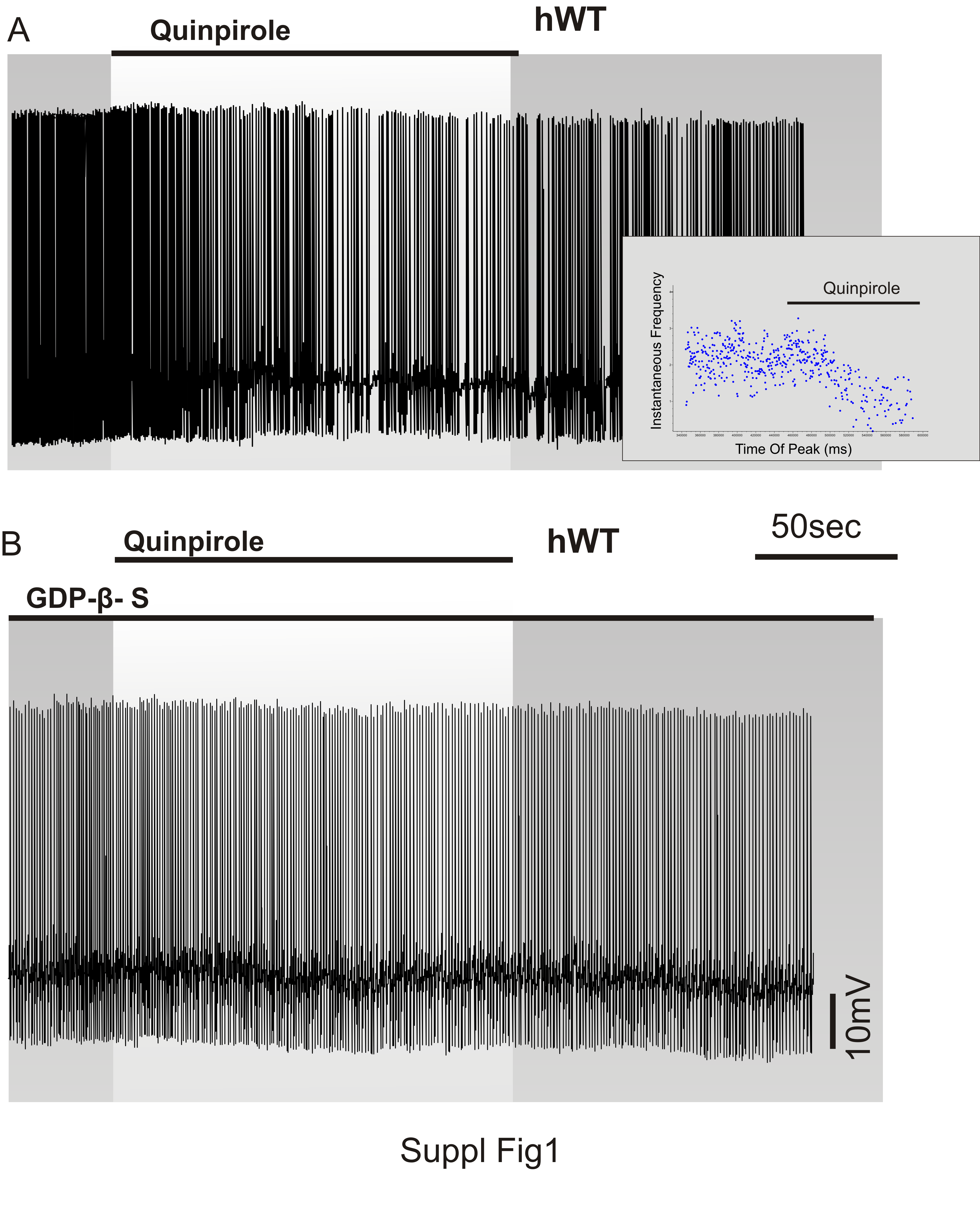

Supplement: Figure S1 — Representative traces of cholinergic interneurons recorded in perforated patch-clamp mode. A. In hWT mice bath-application of quinpirole (10 μM 2-4 min) caused a small reduction of firing rate activity. Time-course plot (inset) shows that quinpirole induced a small reduction of instantaneous firing frequency. B. In hWT animals, D2R activation did not produce any significant effect on membrane potential or firing activity when GDP-β-S (10 mM) was added to the intracellular recording solution. (TIF) [file pone.0024261.s001.tif]
